# Good clinical outcomes after patellar cartilage repair with no evidence for inferior results in complex cases with the need for additional patellofemoral realignment procedures: a systematic review

**DOI:** 10.1007/s00167-021-06728-z

**Published:** 2021-09-12

**Authors:** Daniel Burger, Matthias Feucht, Lukas N. Muench, Philipp Forkel, Andreas B. Imhoff, Julian Mehl

**Affiliations:** 1 Department for Orthopedic Sports Medicine, Technical University Munich, Ismaninger Str. 22, 81675 Munich, Germany; 2 Department of Orthopaedic Surgery, Paulinenhilfe, Diakonieklinikum, Stuttgart, Germany

**Keywords:** Knee, Cartilage, Patella, Patellofemoral, Cartilage repair, Alignment, Review

## Abstract

**Purpose:**

Focal, patellar cartilage defects are a challenging problem as most cases have an underlying multifactorial pathogenesis. This systematic review of current literature analysed clinical results after regenerative cartilage repair of the patella with a special focus on the assessment and treatment of existing patellofemoral malalignment.

**Methods:**

A systematic review was conducted to identify articles reporting clinical results after cartilage regenerative surgeries of the patella using the PubMed and Scopus database. The extracted data included patient-reported outcome measures (PROMS) and whether cartilage repair was performed alone or in combination with concomitant surgeries of underlying patellofemoral co-pathologies. In cases of isolated cartilage repair, specific exclusion criteria regarding underlying co-pathologies were screened. In cases of concomitant surgeries, the type of surgeries and their specific indications were extracted.

**Results:**

A total of 35 original articles were included out of which 27 (77%) were cohort studies with level IV evidence. The most frequently used technique for cartilage restoration of the patella was autologous chondrocyte implantation (ACI). Results after isolated cartilage repair alone were reported by 15 (43%) studies. Of those studies, 9 (60%) excluded patients with underlying patellofemoral malalignment a priori and 6 (40%) did not analyse underlying co-pathologies at all. Among the studies including combined surgeries, the most frequently reported concomitant procedures were release of the lateral retinaculum, reconstruction of the medial patellofemoral ligament (MPFL), and osteotomy of the tibial tubercle. In summary, these studies showed lower preoperative PROMS but similar final PROMS in comparison with the studies reporting on isolated cartilage repair. The most frequently used PROMS were the IKDC-, Lysholm- and the Modified Cincinnati Score.

**Conclusion:**

This comprehensive literature review demonstrated good clinical outcomes after patellar cartilage repair with no evidence of minor results even in complex cases with the need for additional patellofemoral realignment procedures. However, a meaningful statistical comparison between isolated patellar cartilage repair and combined co-procedures is not possible due to very heterogeneous patient cohorts and a lack of analysis of specific subgroups in recent literature.

**Level of evidence:**

Level IV.

## Introduction

Focal cartilage defects of the knee are a common problem, especially in young and active patients as they can lead to pain, swelling and altered joint function [30]. Additionally, there is evidence that these defects are associated with an increased risk of early osteoarthritis over time [12, 64].

Although the general benefit of cartilage regenerative surgeries in the knee has been proven, the patellofemoral joint has often been considered a problematic location by many previous studies [16, 49, 51, 55, 63]. A recent systematic review by Hinckel et al. including 59 articles, did not confirm these concerns. This review showed that cartilage restoration of the patellofemoral joint led to improved clinical outcomes along with low complication rates [32]. However, the authors also reported that lesions at the patella may lead to worse results in direct comparison with those at the trochlea.

Focal cartilage defects of the patella are challenging as in most cases a multifactorial pathogenesis is underlying. It is known that patella dislocations lead to cartilage defects in up to 95% of cases and the risk of (osteo-)chondral flake fractures is reported in up to 58% of patients [41, 50, 58]. Since the risk for re-dislocation of the patella is almost 50% within the first 2 years, additional patella stabilisation is necessary, if surgical therapy of the cartilage defect is planned [4]. Consequently, predisposing factors for patella instability must be analysed and considered when appropriate [72]. Factors include trochlea dysplasia, patella alta, increased tibial tuberosity–trochlea groove (TTTG) distance, genu valgum and increased femoral torsion. [1, 4, 14, 33, 71]

However, even without history of patella dislocation, cartilage defects of the patellofemoral joint are highly associated with co-pathologies, whereas trochlea dysplasia, patella alta and increased lateral patella tilt seem to be particularly predisposing [3, 44]. Therefore, also in these cases, possible co-pathologies must be properly analysed and considered carefully if surgical treatment of patellar cartilage defects is planned. Additionally, for correct interpretation of clinical results after regenerative cartilage procedures at the patella, information regarding the presence and, if applicable, about the surgical treatment of these co-pathologies is necessary. To date, the influence of concomitant procedures addressing patellofemoral stability and alignment in combination with surgical cartilage restoration at the patella is still unclear.

The purpose of the present study was to perform a systematic literature review of clinical trials investigating the results after regenerative cartilage repair of the patella. Among these studies, a special focus was set on the analysis and treatment of preoperative co-pathologies.

It was hypothesised that in most of the included studies, patients with relevant co-pathologies were excluded a priori or a proper presentation of co-pathologies did not exist. Additionally, it was hypothesised that additional treatment of co-pathologies would lead to similar results in comparison with isolated regenerative cartilage therapy at the patella.

## Materials and methods

### Search details

A comprehensive literature search to identify articles reporting clinical results after cartilage regenerative surgeries at the patella was conducted according to the PRISMA statement (Preferred Reporting Items for Systematic Reviews and Meta-Analysis) [40]. The PubMed database and the Scopus database were used for this literature research.

### Inclusion and exclusion criteria

Inclusion criteria to qualify for this systematic review were:

1. Clinical trials reporting results after regenerative cartilage therapy for focal cartilage defects at the patella.
2. Results reported by means of patient-reported outcome measures (PROMs).
3. Level of evidence (LOE) 1–4.
4. English language.

Exclusion criteria were:

1. Publication dates earlier than the year 2000.
2. Follow-up less than 12 months.
3. Less than 5 patients with cartilage lesions located at the patella.
4. No outcomes reported separately for patients with cartilage lesions located at the patella.
5. Only children and adolescents included.
6. Other systematic reviews and meta-analyses.

### Search strategy

Different combinations of the following keywords were used for the initial data base search: cartilage repair, cartilage restoration, cartilage transplantation, cartilage implantation, microfracture, microfracturing, osteochondral autologous transfer, OATS, mosaicplasty, osteochondral allograft transplantation, autologous chondrocyte implantation, ACI, MACI, patella, patellar, patellofemoral. The search was performed in April 2020. All abstracts of the identified publications were judged for inclusion suitability primarily by authors DB and JM. If the abstract showed any inclusion criteria, the entire paper was read. All authors performed the analysis of the articles based on the inclusion and exclusion criteria and all authors had to agree to include or exclude an article.

If two separate studies had the same authors and intervention but had different follow-up, then only the study with the longer follow-up was included for the outcome analysis.

### Study quality

The quality of the included studies was analysed by means of the Methodological Index for Non-Randomised Studies (MINORS), which consists of eight items for non-comparative studies and four additional items for comparative studies [62]. A maximum of 2 points can be assigned to each item, resulting in a maximum score of 16 points for non-comparative studies and 24 points for comparative studies. The assessment was performed independently by two reviewers (DB, JM) and the final score was determined by consensus. Additionally, the level of evidence (LOE) of the included studies was registered.

### Data extraction and analysis

For all included studies, the extraction of data included: the first author’s name, publication year, journal, study design, LOE, MINORS, number of cases with patellar cartilage defects, patients’ age, follow-up time, defect size, and surgical technique. In the case of comparative studies, the definition of the study groups was documented. If subgroups of patients with patellar cartilage defects were defined and the demographic data and results were given separately, only this data was extracted. As already mentioned above, studies with no separate data for patellar defects were excluded from the analysis. This also applied for studies that combined patellofemoral cartilage defects into one study group.

For all included studies, the used PROMS were noted and the corresponding results were analysed. If a comparison of preoperative PROMS with PROMS at final follow-up was performed, the p-value representing a possible significant difference was documented. If a comparison with preoperative PROMS was not performed or if a *p* value was not given, the main outcome of the studies was extracted as a short summary.

Furthermore, all included studies were analysed whether isolated cartilage repair alone was performed, or concomitant surgeries of underlying co-pathologies were performed in combination with cartilage repair.

In the case of isolated cartilage repair, the specific inclusion and exclusion criteria were analysed to further characterise the study cohort and to evaluate if patients with typical co-pathologies were excluded.

In the case of concomitant surgeries, the type of surgeries and, if given, the specific indications for these surgeries were extracted. For studies that directly compared patients with and without concomitant surgeries, the results were extracted for each group separately.

### Statistical analysis

The extracted quantitative parameters (age, follow-up time, defect size and results of the PROMs) were given as mean ± standard deviation (SD), when provided in the articles. Otherwise, alternative values like median or range were extracted.

Due to the high statistical and methodological heterogeneity of the included studies, a meta-analysis comparing the results between patients with and without concomitant surgeries was not possible. Instead, a narrative description and comparison of the clinical results was performed.

## Results

### Search results and study design

**Fig. 1:**
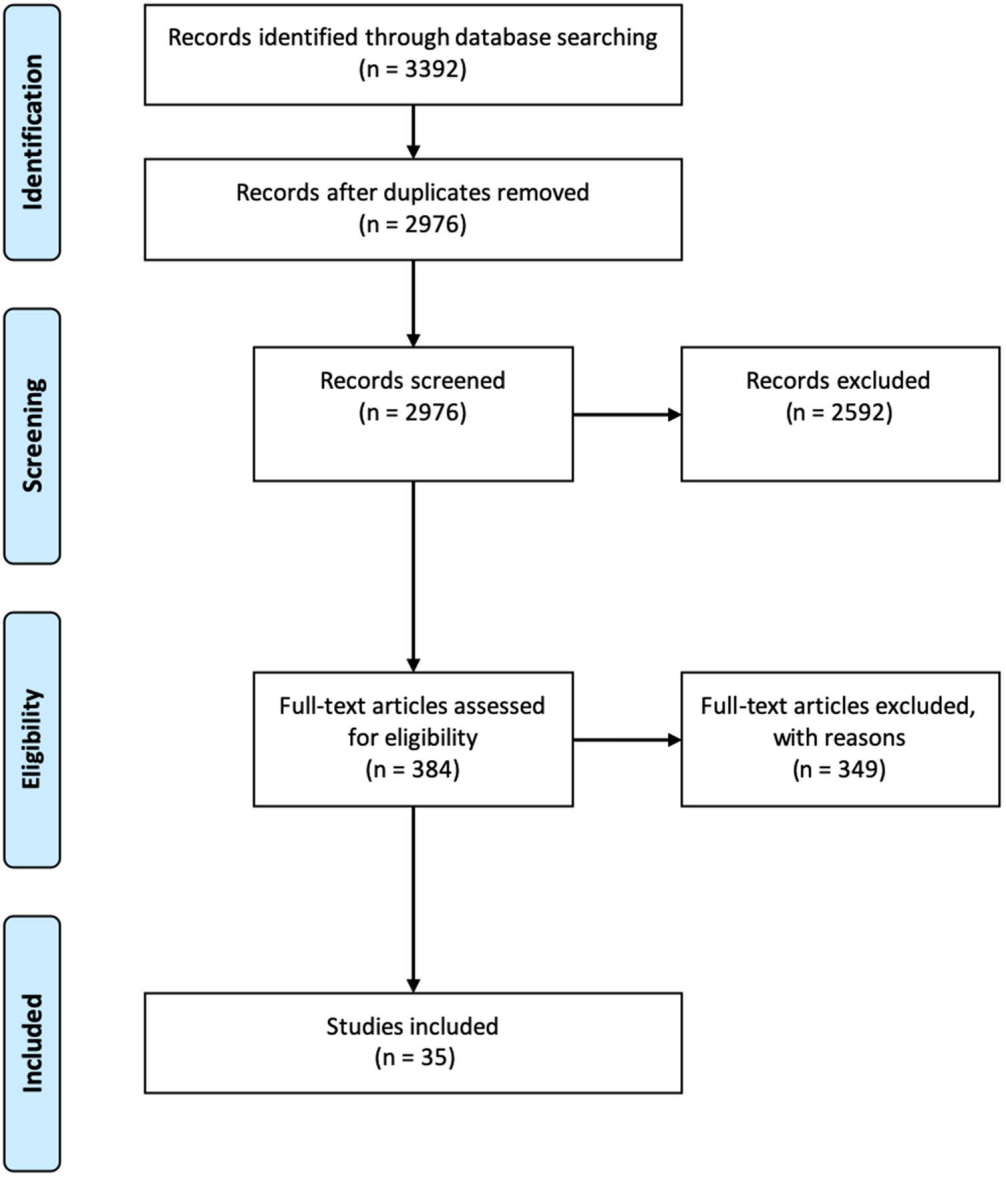
PRISMA flow chart of the study inclusion

After screening for eligible studies, a total of 35 original articles were identified and included in this systematic review (Fig. 1, Table 1). With 27 studies (80%), the vast majority were prospective or retrospective cohort studies with level IV evidence. The mean MINORS score was 13.3 of 16 (range from 10 to 16) for non-comparative studies and 20.1 of 24 (range from 16 to 23) for comparative studies. The number of reported cases ranged from 6 to 110, the mean age of the included patients ranged from 15 to 39.2 years, and the mean follow-up time ranged from 24 to 153 months. The most frequently used techniques for cartilage restoration were autologous chondrocyte implantation (ACI) in 48.6% and autologous osteochondral transplantation (AOT) in 22.9% of cases. The range of retropatellar cartilage defect sizes in studies using the ACI technique was 2.8–6.4 cm^2^ and for AOT 1.16–1.6 cm^2^.

**Table 1:** Study design, study quality, and summary of clinical and operative topics of all included 35 studies

Authors (year)/journal	Study design/LOE/MINORS	Cases (N)	Age (years) mean	FU (months) mean	Study groups	Patellar cartilage defect size mean (cm^2^)	Technique
Akgün, Akpolat [2] J Orthop Surg	Retrospective cohort LOE 4/MINORS 13/16	14	29.7	44.4	No	1.32	AOT
Astur et al. [5] J Bone Joint Surg Am	Prospective cohort LOE 4/MINORS 16/16	33	37.6	30.2 (median)	No	n.i	AOT
Astur et al. [6] Knee Surg Sports Traumatol Arthrosc	Prospective cohort LOE 4/MINORS 14/16	20	26–45 (range)	24	No	1.16	AOT
Biant et al. [8] Am J Sports Med	Prospective cohort LOE 4/MINORS 16/16	36	29.7	> 120	Subgroup patella	4.49	ACI-C (85x)
							ACI-P (19x)
Bouwmester et al. [9] J Orthop Res	Prospective comparative LOE 2/MINORS 16/24	10	28.9 (SD 7.8)	132.2 (SD 57.4)	Perichondrium Tx	2.8	Perichondrium Tx
					Debridement + drilling		Debridement + drilling
Chadli et al. [11] Int Orthop	Retrospective cohort LOE 4/MINORS 11/16	8	15.0	28.6	No	0.97	Autologous osteochondral mosaicplasty
Cohen et al. [13] Rev Bras Ortop	Prospective cohort LOE 4/MINORS 12/16	17	38.1 (SD 13.4)	19.8	No	n.i	AOT
Figueroa et al. [19] Knee	Prospective cohort LOE 4/MINORS 15/16	10	20.2	37.3	No	1.2	AOT
Filardo et al. [20] Am J Sports Med	Prospective comparative LOE 2/MINORS 23/24	28	29.3 (SD 8.9)	60	Patella (28)	All: 3.0	MACI
					Trochlea (17)	Patella: 2.8	
					Both (4)		
Gaweda et al. [22] Int Orthop	Retrospective comparative LOE 3/MINORS 18/24	Group 1: 19	Group 1: 25.5	24	Group 1: realignment	All: > 1	AOT
		Group 2: 30	Group 2: 21.7		Group 2: realignment + AOT		
Gigante et al. [23] Knee Surg Sports Traumatol Arthrosc	Prospective cohort LOE 4/MINORS 15/16	14	31 (median)	36	No	4 (median)	MACI
Gillogly et al. [24] Am J Sports Med	Retrospective cohort LOE 4/MINORS 12/16	25	31.0 (SD 7.0)	90.7 (SD 27.6)	No	6.4	ACI-P
Gobbi et al. [26] Am J Sports Med	Prospective cohort LOE 4/MINORS 15/16	22	30.5	24	No	4.7	MACI
Gomoll et al. [27] Am J Sports Med	Prospective cohort LOE 4/MINORS 13/16	110	33.0 (SD 10.1)	90 (SD 31.7)	No	5.4	ACI-P
Gracitelli et al. [28] Am J Sports Med	Retrospective cohort LOE 4/MINORS 12/16	28	33.7	116.4 (SD 7.5)	No	10.1	OCA
Hangody et al. [29] Am J Sports Med	Prospective cohort LOE 4/MINORS 11/16	18	24.0	115.2	No	2.4	Mosaicplasty
Henderson, Lavigne [31] Knee	Retrospective comparative LOE 3/MINORS 23/24	Group A: 22	Group A: 32.1	Group A: 26.2	Group A: with realignment	Group A: 2.92	ACI-P
		Group B: 22	Group B: 25.1	Group B: 28.9	Group B: no realignment	Group B: 3.22	
Joshi et al. [35] Am J Sports Med	Prospective cohort LOE 4/MINORS 14/16	10	33.3	24	No	2.64	TruFit (synthetic osteochondral scaffold plug)
Kreuz et al. [38] Osteoarthritis Cartilage	Retrospective cohort LOE 4/MINORS 13/16	18	28.4 (SD 8.8)	36	No	5.7	ACI-P
Kreuz et al. [37] Am J Sports Med	Prospective cohort LOE 4/MINORS 15/16	13	35.2 (SD 10.7)	48	No	4.69	ACI (BioSeed-C)
Kusano et al. [39] Knee Surg Sports Traumatol Arthrosc	Retrospective cohort LOE 4/MINORS 14/16	20	39.2 (SD 2.8)	29.3 (SD 2.3)	No	4.4	AMIC
Macmull et al. [42] Int Orthop	Retrospectve comparative LOE 4/MINORS 19/24	48	34.8	40.3	Group 1: ACI-C (25)	Group 1: 4.73	MACI
					Group 2: MACI (23)	Group 2: 4.76	ACI-C
Minas and Bryant [45] Clin Orthop Relat Res	Prospective cohort LOE 4/MINORS 14/16	8	35.0	47.5	No	4.34	ACI-P
Nho et al. [47] Am J Sports Med	Retrospective cohort LOE 4/MINORS 15/16	22	30.0 (SD 12.0)	28.7	No	1.6	AOT
Niemeyer et al.[ 49] Arch Orthop Trauma Surg	Restrospective cohort LOE 4/MINORS 12/16	70	34.3 (SD 10.1)	38.4 (SD 15.6)	ACI-P	4.41	ACI-P
					ACI-C		ACI-C
					MACI		MACI
Niemeyer et al. [48] Arch Orthop Trauma Surg	Retrospective comparative LOE 3/MINORS 22/24	45	33.5 (SD 8.88)	60	45 Patella	5.4	MACI
					28 Fem. condyle		
Perdisa et al. [52] Am J Sports Med	Prospective cohort LOE 4/MINORS 14/16	34	30.0 (SD 10.0)	24	No	2.1	Cell-free biphasic collagen-hydroxy apatite osteo-chondral scaffold
Peterson et al. [55] Am J Sports Med	Retrospective cohort LOE 4/MINORS 10/16	34	34.0	153.6	No	6.1	ACI-P
Sadlik et al. [57] J Knee Surg	Prosepective cohort LOE 4/MINORS 13/16	12	36.0	38	No	2.5	AMIC
Spahn, Kirschbaum [65] Knee Surg Sports Traumatol Arthrosc	Retrospective comparative LOE 4/MINORS 20/24	42	Group A:	Group A:	Group A:	Group A: 31.4 (diameter mean, mm)	Abrasive arthroplasty
			27.3	38.4	Abrasive		Periostal arthroplasty
			(SD 6.6)	(SD 1.0)	arthroplasty (25)		
			Group B:	Group B:	Group B:	Group B: 30.6 (diameter mean, mm)	
			25.7	37.3	Periostal		
			(SD 6.5)	(SD 1.1)	arthroplasty (17)		
Teo et al. [69] Clin Orthop Relat Res	Retrospective cohort LOE 4/MINORS 12/16	23	16.8	72	No	n.i	ACI-P (20x) BMSCs implantation with periost patch (3x)
Visona et al. [73] Orthop Traumatol Surg Res	Retrospective cohort LOE 4/MINORS 12/16	6	20.5 (SD 9.2)	26	No	0.88	Mosaicplasty
von Keudell et al. [74] Cartilage	Prospective cohort LOE 4/MINORS 14/16	30	32.0 (SD 10.0)	88	No	4.7	ACI-P, ACI-C
Yonetani et al. [76] J Orthop Case Rep	Retrospective cohort LOE 4/MINORS 14/16	6	38.0 (SD 8.0)	51	No	1.24	AOT

*LOE* Level of evidence, *FU* Follow-up, *SD* Standard deviation, *n.i* no information, *AOT* Autologous osteochondral transplantation, *ACI-C* ACI using collagen scaffold, *ACI-P* ACI using periostal flap, *Tx* Transplantation, *MACI* Matrix associated ACI, *OCA* Osteochondral allograft, *AMIC* Autologous matrix-induced chondrogenesis, *BMSC* Bone marrow-derived mesenchymal stem cell

### Isolated cartilage repair

In this systematic review, 15 studies (43%) reported on patellar cartilage repair without any concomitant surgeries. Detailed information about the inclusion/exclusion criteria and the main results of these 15 studies are presented in Table 2 (online addition). The analysis showed that underlying pathologies were not reported at all (6 studies) or patients with appropriate co-pathologies were excluded a priori (9 studies). The most often reported exclusion criteria of co-pathologies were tibio-femoral varus/valgus malalignment (6x) and patellofemoral malalignment, such as patella alta or baja (3x), increased patellar tilt (3x), increased patellar shift (1x), increased TTTG > 15 mm (1x) or trochlea dysplasia (1x). Eleven out of the 15 surveys with isolated patellar cartilage repair (73%) reported at least one significantly improved patient-reported outcome measure (PROMs) of which as many as 16 different scores were assessed. The most often significantly improved PROMs postoperatively reported were the Lysholm- (5x) and the Kujala-score (3x). On average, the mean values improved from 61.6 (range 42.7–73.8) to 90.9 (range 67.6–95) for the Lysholm score and from 49.3 (range 44.9–54.8) to 76.8 (range 75.2–78.4) for the Kujala score.

**Table 2:** (online addition): Studies without surgical treatment of concomitant pathologies

Authors (year)/journal	Inclusion criteria	Exclusion criteria	PROMS			*p* value
			Score	preop (mean)	postop (mean)	
Akgün, Akpolat [2] J Orthop Surg	Age 18–55 years, defect size > 0.8 cm^2^, osteochondral lesion patella, symptoms > 6 months	Alignment problems (patellar height, varus-valgus), chondral lesion < 0.8 cm^2^	VPS	75.5	17.57	*p *< 0.01
			Lysholm	44.57	80	*p *< 0.01
			Kujala	48.21	78.42	*p *< 0.01
Astur et al. [5] J Bone Joint Surg Am	Age < 60 years, anterior knee pain, patellar chondral lesion, grade 3 or 4, diameter 1–2.5 cm	Diameter < 1 and > 2.5 cm, patella tilt, patella alta or baja, TTTG > 15 mm, ACL injury, meniscal tear, infection, systemic inflammatory disease	Lysholm	57.27	80.76	*p *< 0.05
			Fulkerson	54.24	80.42	*p *< 0.05
			Kujala	54.76	75.18	*p *< 0.05
			SF-36	see study details		
Astur et al. [6] Knee Surg Sports Traumatol Arthrosc	Age < 45 years, traumatic injury, patellar chondral lesion, grade 3 or 4, diameter 1–2.5 cm	Diameter < 1 and > 2.5 cm, patella tilt, patella alta or baja, TTTG > 15 mm, ACL injury, meniscal tear	VAS	7.1	2.4	*p *< 0.05
			Tegner	n.i		
			Kujala	44.9	76.9	*p *< 0.001
Biant et al. [8] Am J Sports Med	Symptomatic isolated cartilage defect	Limb malalignment, ligament deficiency, osteoarthritis, inflammatory arthritis, defect depth > 5 mm	Mod. Cincinnati knee score	40	79	n.i
			Stanmore/Bentley score	3	1.3	
			VAS	6.4	2	
Bouwmester et al. [9] J Orthop Res	Age < 40 years, no previous drilling, isolated defect	Osteoarthritis > grade 2	HSSS	80.8	92.2	n.i
			VAS walk		1.8	
			VAS rest		0.9	
Chadli et al. [11] Int Orthop	Clinical symptoms: pain, crepitus, hydarthrosis, locking, MRI: OCD grade 3 or 4	n.i	IKDC	49.9	86.1	*p *< 0.001
			Lysholm	53.8	88.5	*p *< 0.001
			Tegner	4.5	6.2	*p *= 0.02
Figueroa et al. [19] Knee	Age < 45 years, patellar chondral lesion grade 4, defect size < 2.5 cm^2^	Multiligamentous lesions, concomitant ACL-reconstruction, other cartilage lesions than patella, defect size > 2.5 cm^2^	Lysholm	73.8	95	*p *< 0.05
			IKDC	na	95	
Joshi et al. [35] Am J Sports Med	Patellofemoral pain, full-thickness patellar cartilage defect	Age < 15 years and > 50 years, patellofemoral malalignment (> 10° tilt), tibiofemoral malalignment (> 10°), chondral lesions other location than patella	KOOS	64.7	69.9	n.i
			VAS	7.9	6.9	
			SF-36	n.i	61.3	
Kreuz et al. [38] Osteoarthritis Cartilage	ACI-P for outerbridge grade 3 or 4 defects	Acute trauma, varus or valgus malalignment > 5°, limits in knee extension, limits in knee flexion < 130°, patellofemoral malalignment with med. or lat.l shift > 5 mm, ACL- or MCL instability, Meniscal pathologies, I.a. corticosteorid injections < 1 month	Cincinnati score patella	3.67	2.22	*p *< 0.05
			ICRS score patella	3.72	2.5	*p *< 0.05
Macmull et al.[42] Int Orthop	Chondral or osteochondral defect secondary to chondro-malacia patellae	n.i	VAS all	6.42	4.5	*p *< 0.001
			VAS Group 1	6.32	5	*p *= 0.017
			VAS Group 2	6.52	3.96	*p *< 0.001
			Mod. Cinc. score all	45.13	54.81	*p *= 0.01
			Mod. Cinc. score Group 1	42.12	48.76	n.s
			Mod. Cinc. score Group 2	48.39	61.39	*p *< 0.001
			Bentley all	2.92	2.27	*p *< 0.001
			Bentley Group 1	3.04	2.44	*p *= 0.013
			Bentley Group 2	2.78	2.09	*p *< 0.001
Niemeyer et al. [49] Arch Orthop Trauma Surg	Retropatellar cartilaginous damage	Trochlea dysplasia, varus or valgus deformity > 5°	Cincinnati sports activity	34.44	61.5	*p *< 0.001
			Lysholm	n.i	73	
			IKDC	n.i	62	
Niemeyer et al. [48] Arch Orthop Trauma Surg	Age 18–50 years, isolated, chondral or osteo-chondral defects ICRS 3 or 4, defects size 4–10 cm^2^ after debridement, OCD with max depth 6 mm	Radiological signs of osteoarthritis, valgus or varus malalignment > 5°, previous treatment with ACI, and many more	KOOS overall patella	54.6	82.6	*p *= 0.0099
			KOOS Pain Patella	61.2	88.3	*p *< 0.001
			KOOS Symptoms patella	69.9	87.6	*p *< 0.001
			KOOS ADL patella	71.4	91.4	*p *< 0.001
			KOOS Sport patella	43.1	76	*p *< 0.001
			KOOS QOL patella	28.1	70.6	*p *< 0.001
Peterson et al. [53] Am J Sports Med	Chondral lesion outerbridge grade 3 or 4, severe symptoms, poor results according to clinical grading system of Brittberg et al	n.i	Cincinnati patella	1.6	6.6	*p *< 0.001
			Brittberg VAS patella	68.1	27.8	*p *< 0.001
			Tegner–Wallgren patella	5.5	8.8	*p *< 0.001
Spahn, Kirschbaum [65] Knee Surg Sports Traumatol Arthrosc	Cartilage defect patella Outer-bridge grade 3 or 4	Patellar malalignment, residual complaints after patella fracture or patella dislocation	Lysholm Group A	36.1	42.5	*p *< 0.05
			Lysholm Group B	42.7	67.6	*p *< 0.05
			Tegner Group A	5.5	2.7	*p *< 0.05
			Tegner Group B	5.5	4.9	n.s
			VAS Group A	80.4	84.4	n.s
			VAS Group B	73.4	25.9	*p *< 0.05
Yonetani et al. [76] J Orthop Case Rep	Focal patellar cartilage defect, normal patellofemoral alignment, failure of conservative treatment > 6 months	n.i	Lysholm	67	90	n.i

*VPS* Visual pain scale, *VAS* Visual analogue scale, *n.i* no information, *HSSS* Hospital for Special Surgery Knee Score, *OCD* Osteochondritis dissecans, *IKDC* International Knee Documentation Committee, *KOOS* Knee Osteoarthritis Outcome Score, *ADL* Activities of daily living, *QO* Quality of Life, *ICRS* International Cartilage Regeneration and Joint Preservation Society, *n.s* not significant, *TTTG* tibia tuberosity-trochlea groove distance, *ACL* Anterior cruciate ligament, *MCL* Medial collateral ligament, *ACI-P* ACI using periostal flap

### Concomitant surgeries

In 20 of the 35 studies (57%), patients with the need for additional procedures due to underlying patellofemoral co-pathologies were included. Detailed information about the concomitant surgeries, their indications and the main results of these 20 studies are presented in Table 3 (online addition). Among these studies, eight (40%) reported on either soft-tissue or bony realignment procedures, and six (30%) on both soft-tissue and bony realignment procedures. Another four studies (20%) included a mixture of patients with additional soft-tissue or bony realignment procedures or the combination of both. In one study, cartilage repair was combined with soft-tissue procedures only, and in another study, it was combined with bony realignment procedures only. In 17 studies, the results of both isolated cartilage repair and cartilage repair combined with surgery of concomitant pathologies were merged. There were three studies that only reported on combined surgical approaches. [22–24]

**Table 3:** (online addition): Studies including surgical treatment of concomitant pathologies

Authors (year)/journal	Concomitant surgeries	Indications for concomitant surgery	PROMS			*p* value
			Score	preop (mean)	postop (mean)	
Cohen et al. [13] Rev Bras Ortop	MPFL reconstruction (1x), Lat. release (6x)	Traumatic patellar dislocation, excessive lat. patellar tilt	Lysholm	54.59	75.76	*p *< 0.05
			Fulkerson	52.53	78.41	*p *< 0.05
			Kujala	49.82	73.47	*p *< 0.05
			SF-36	see study details		see study details
Filardo et al. [20] Am J Sports Med	Lat. release (13x) HTO (6x)	n.i	IKDC	36.2	69.7	IKDC with sig. improvement
			Kujala	na	81.5	
			EQ-VAS	na	81.9	
			Tegner	Improvement: 3.9		
Gaweda et al. [22] Int Orthop	Combination of proximal (lat release, VMO transfer) and distal (TTO) extensor realignment	Recurrent patellar dislocation or subluxation	Marchall score	36.3	46.2	Mean score improved faster than in the control group
Gigante et al. [23] Knee Surg Sports Traumatol Arthrosc	TTO	Type 2 patellofemoral malalignment according to Fulkerson, TTTG > 20 mm	Kujala	52	88.5	*p *= 0.001
			Lysholm	55	92.5	*p *= 0.001
			Tegner	1	4	*p *= 0.001
			Mod. Cincinnati rating scale	2	8	*p *= 0.001
Gillogly et al. [24] Am J Sports Med	TTO (25x), trochleoplasty (4x), Lat. release (25x), Med. imbrication or reefing	Failure to centralise patella in the trochlea by > 45°, increased Q-angle, arthroscop. lat. patellar maltracking, Recurr. dislocations, flat or convex trochlear entrance, increased patellar tilt, patients with excessive laxity	Mod. Cincinnati rating scale	3	7	*p *< 0.0001
			Lysholm	40.2	79.3	*p *< 0.0001
			IKDC	42.5	75.7	*p *< 0.0001
			SF-12 PCS	41.2	47.6	*p *= 0.002
			SF-12 MCS	48.1	60.7	*p *= 0.0001
Gobbi et al. [25] Am J Sports Med	Patellofemoral realignment (2x), Lat. release (3x), meniscectomy (3x),	n.i	IKDC	43.2	73.6	*p *< 0.0001
Gomoll et al. [27] Am J Sports Med	TTO (75x), Lat. release (45x), trochleaplasty (5x), Vastus med. advancement (22x), MPFL-reconstruction (1x)	History of patellar instability, Patellar maltracking, TTTG > 15 mm, large, uncontained or bipolar defects, decreased patellar mobility, trochleadysplasia	SF-12 PCS	38.6	44.1	*p *= 0.001
			SF-12 MCS	49.7	53.5	n.s
			IKDC	40.2	69.4	*p *< 0.0001
			Mod. Cincinnati knee score	3.2	6.2	*p *< 0.0001
			WOMAC	50.4	29.6	*p *< 0.0001
			KSS Knee	61.8	85.2	*p *< 0.0001
			KSS Function	58.5	72.7	*p *< 0.0001
Gracitelli et al. [28] Am J Sports Med	Lat. release (7x), Vastus med. imbrication (1x), TTO + MPFL-reconstruction (3x), TTO only (3x)	Sign. malalignment or instability of PFJ in physical examination	Merle d’Aubigne-Postel score	12	15.2	*p *= 0.003
			IKDC	36.5	66.5	*p *= 0.003
			KS-F	64.6	80.5	*p *= 0.003
Hangody et al. [29] Am J Sports Med	Lat. release (11x), TTO (3x), meniscus resection (2x)	n.i	HSSS	57	71	n.s
Henderson et al. [31] Knee	Group A: Lat. release + TTO + MPFL tensioning (22x)	Lateralisation of the patella during first 45° of flexion	IKDC all	42.3	68.1	*p *< 0.006
			Mod Cincinnati score all	3.4	6.5	*p *< 0.05
			Cincinnati Group A	Improvement.: 4.46		Group A better (*p *< 0.001)
	Group B: ACI-P only		Cincinnati Group B	Improvement.: 1.73		
			IKDC Group A	Improvement.: 36.2		Group A better (*p *< 0.05)
			IKDC Group B	Improvement.: 22.3		
Kreuz et al. [37] Am J Sports Med	Patella balancing (2x), HTO (10x), microfracturing of secondary lesions (5x), subchondral bone grafting (2x), ACL-reconstruction (6x)	Med. or lat. shift > 5 mm, varus/valgus malalignment > 5°	ICRS patella	4	2.1	n.i
			IKDC patella	44.1	68.2	
			KOOS pain patella	62.4	75.2	
			KOOS symptoms patella	70.8	73.3	
			KOOS ADL patella	67.8	81.5	
			KOOS sport patella	11.2	52.7	
			KOOS QOL patella	29.5	54.4	
			Lysholm	51.2	78.2	
Kusano et al. [39] Knee Surg Sports Traumatol Arthrosc	TTO + lat. release (18x)	Patellar maltracking	IKDC patella	51	74	*p *= 0.0025
			Lysholm patella	58	85	*p *< 0.0001
			Tegner patella	3	4	n.s
			VAS patella	6	2	*p *= 0.0004
Minas et al. [45] Clin Orthop Relat Res	TTO + lat. release (5x)	Patellofemoral malalignment (patellar subluxation or tilt)	SF-36 PCS patella	32.84	40.06	*p *= 0.02
			SF-36 MCS patella	45.1	43.99	n.s
			KSS patella	47.13	71.88	*p *= 0.01
			KSS function patella	49.38	70.63	*p *= 0.01
			WOMAC patella	56.75	34.88	*p *= 0.02
			Mod. Cincinnati score patella	3.63	5.13	*p *= 0.03
Nho et al. [47] Am J Sports Med	Lat. release (13x), TTO (9x), proximal realignment (3x)	Patellofemoral malalignment, surgeons preference	IKDC all	47.2	74.4	*p *= 0.028
			ADL all	60.1	84.7	*p *= 0.022
			SF-36 all	64	79.4	n.s
			IKDC (AOT + TTO)	54.3	64.9	n.s
			ADL (AOT + TTO)	66	81.6	n.s
			SF-36 (AOT + TTO)	64.7	70.7	n.s
Perdisa et al. [52] Am J Sports Med	TTO (9x), Lat. release (1x), MPFL-reconstruction (1x), removal of calcifications (3x), MAT (1x), patellar tendon repair (1x)	n.i	IKDC	39.5	67.6	*p *< 0.001
			Tegner	1.8	3.3	*p *< 0.001
Peterson et al. [55] Am J Sports Med	TTO, med. soft-tissue plication, lat release + trochleaplasty (21x), TO, med. soft-tissue plication, lat. release (7x) Med. soft-tissue plication + trochleaplasty (1x) Lat. release + trochleaplasty (1x) HTO (2x)	Patellofemoral malalignment	Lysholm patella	69	66	n.s
			Tegner–Wallgren patella	7.4	8.1	n.s
			KOOS pain patella	n.i	69.7	
			KOOS symptoms patella	n.i	67.5	
			KOOS ADL patella	n.i	81.3	
			KOOS sports patella	n.i	41.1	
			KOSS QOL patella	n.i	48.2	
			Mod. Cincinnati patella	n.i	5.1	
			Brittberg–Peterson patella	50.1	49.2	n.s
Sadlik et al. [57] J Knee Surg	TTO (2x), MPFL (2x), HTO (1x)	n.i	KOOS	50.3	90.1	*p *< 0.01
			IKDC	37.4	79.4	*p *< 0.01
			VAS	7.8	2.3	*p *< 0.01
Teo et al. [69] Clin Orthop Relat Res	TTO (Elmslie–Trillat) (4x), Roux–Goldthwaite (2x)	Increased TTTG > 15 mm and/or increased patellar tilt > 20°	IKDC	45	75	*p *< 0.001
			Lysholm	50	70	*p *< 0.001
			Tegner	2.5	4	*p *< 0.001
Visona et al. [73] Orthop Traumatol Surg Res	Sectioning of the patellar retinaculum (2x), TTO + MPFL-reconstruction (1x)	n.i	IKDC	37.2	66.3	n.i
			Lysholm	58.3	85	
			Tegner	3.5	5.7	
von Keudell et al. [74] Cartilage	TTO + soft-tissue balancing (19x), Lat. subvastus release (28x), VMO advancement (23x), TTO + trochleaplasty + proximal soft-tissue balancing (5x)	Lat. maltracking, patellar instability, TTTG > 15 mm, hypoplastic trochlea	SF-36 PCS	40	47	*p *= 0.01
			SF-36 MCS	47	53	*p *= 0.02
			KSS function	55.7	73	*p *< 0.01
			KSS pain	63.9	81.8	*p *< 0.01
			WOMAC	52.2	27.9	*p *< 0.01
			Mod. Cincinnati rating scale	3.1	5.7	*p *< 0.01

*VAS* Visual analogue scale, *SF-36* 36-item Short form Health Survey, *SF-12* 12-item Short Form Health Survey, *PCS* physical component score, *MCS* mental component score, *HSSS* Hospital for Special Surgery Knee Score, *WOMAC* Western Ontario and McMaster Universities Osteoarthritis Index, *IKDC* International Knee Documentation Committee, *KSS* Knee Society Score, *KOOS* Knee Osteoarthritis Outcome Score, *ADL* Activities of daily living, *QOL* Quality of Life, *KS-F* Knee Society function scale, *ICRS* International Cartilage Regeneration and Joint Preservation Society, *TTTG* tibia tuberosity-trochlea groove distance, *MPFL* Medial patellofemoral ligament, *HTO* High tibial osteotomy, *VMO* Vastus medialis obliquus, *TTO* Tibial tuberosity osteotomy, *ACI-P* ACI using periostal flap, *ACL* Anterior cruciate ligament, *MAT* Meniscal allograft transplantation, *PFJ* Patellofemoral Joint, n.i no information, *n.s* not significant

The most frequently reported concomitant soft-tissue procedures were the release of the lateral retinaculum (14 studies) and the reconstruction of the MPFL (7 studies). Concomitant bony procedures were osteotomies of the tibial tubercle (17 studies), trochleaplasties (4 studies) and high tibial osteotomies (4 studies).

Among all the studies that included patients after combined surgery, 70% (14 out of 20) reported at least one significantly improved postoperative PROM, of which as many as 19 different scores were assessed. The most often significantly improved PROMs postoperatively reported were the IKDC- (11x), the Modified Cincinnati- (6x) and the Lysholm-scores (4x). On the average, the mean values improved from 41.9 (range 36.2–51) to 72.1 (range 66.3–79.4) for the IKDC-Score, from 3.1 (range 2–3.6) to 6.4 (range 5.1–8) for the Modified Cincinnati score and from 51.9 (range 40.2–69) to 83.1 (range 66–92.5) for the Lysholm-Score.

An isolated analysis of the 3 studies, which reported on combined surgical approaches only, demonstrated statistically significant improvements of all PROMs for 2 studies. [23, 24] The third study showed an improvement of the PROMs, however a statistical analysis was missing. [22]

While most of these 20 studies included heterogeneous patient groups with different combinations of surgical procedures, only one study reported on a homogeneous study group with the same treatment approach for all included patients [23]. Gigante et al. investigated the outcome of 14 patients with MACI for retropatellar chondral lesions in combination with TTO because of patellofemoral malalignment and TTTG > 20 mm. The results showed a significant improvement of all scores after a mean follow-up of 36 months. Additionally, there was only one retrospective comparative study, which directly compared the results of isolated retropatellar cartilage repair with a combination of cartilage repair and the correction of patellofemoral malalignment [31].

The analysis of indications for concomitant surgeries gave a very heterogeneous picture. In 6 out of 20 studies, no specific indications for additional patellofemoral procedures were defined at all. Among the other 14 studies, the indication was based on clinical evaluations and/or radiological values. The most frequently reported indications for additional procedures were patellofemoral malalignment or maltracking (7x), history of patella dislocation or patellofemoral instability (6x), excessive patella tilt (3x) and trochlea dysplasia (3x). In 4 studies, an increased TTTG distance was defined as indication for additional realignment, with 3 studies setting the cut-off value at 15 mm and one study at 20 mm.

## Discussion

The most important finding of the present systematic review was that both isolated patellar cartilage repair alone and patellar cartilage repair combined with patellofemoral alignment correction led to good clinical results.

However, considering the fact that patellofemoral malalignment has been discussed as a risk factor for negative outcomes after patellar cartilage surgery already for several decades[10, 17, 23, 60], a rather surprising finding was that more than 40% of the included studies did not include patients with concomitant surgeries for underlying patellofemoral co-pathologies.

On the other hand, more than half of the identified studies did include patients with the need for additional patellofemoral stabilisation or realignment in combination with cartilage repair at the patella. Most of these studies reported on different combinations of concomitant surgeries and summarised the clinical outcome scores without analysing specific subgroups regarding the surgical approach.

The studies including combined surgical procedures reported good clinical outcomes with a significant improvement of at least one PROM in 14 of 20 studies (70%), while the remaining 6 studies demonstrated an improvement in at least one PROM, but without any statistical significance [29, 47, 55] or a statistical analysis was not available [22, 37, 73]. These results were similar to the studies reporting on isolated cartilage repair at the patella with 11 of 15 studies (73%) observing significant improvements of at least one PROM in the postoperative course. This may support the hypothesis that the need for additional patella stabilisation or realignment is not correlated with worse clinical outcomes. However, due to the very heterogeneous patient cohorts and missing analysis of specific subgroups, comparison between isolated patellar cartilage repair and combined procedures is limited.

Among the 35 included studies, there was only one which directly compared the outcome of isolated chondral repair with chondral repair and simultaneously addressing underlying patellofemoral malalignment[31]. Henderson et al. investigated 22 patients after ACI-P only and 22 patients after ACI-P in combination with lateral release, TTO and MPFL tensioning. Both groups showed improved final follow-up scores with significantly worse results for the ACI-P only group.

The efficacy of cartilage repair surgeries in the patellofemoral joint has been proven by several studies investigating different surgical techniques [15, 18, 32, 67, 68, 75]. However, a recent systematic review concluded that lesions at the patella might lead to worse results in comparison with the trochlea. One reason for this finding may be the fact that anatomic patellofemoral risk factors are more often associated with cartilage defects at the patella in comparison with the trochlea [3]. Because of these etiological and clinical differences between the patellar and trochlear location, only studies reporting outcomes after cartilage repair at the patella were included in the present literature review.

The high prevalence of anatomic risk factors in association with cartilage defects at the patella has been shown by several studies [3, 21, 44]. Therefore, the main focus of the present review was set on how underlying co-pathologies were taken into account when reporting the results after cartilage repair at the patella. Almost half of the included studies reported on isolated cartilage repair surgery at the patella without any additional procedures. In several of these studies, inclusion and exclusion criteria were not adequately reported and it is not clear if patellofemoral risk factors were present among the treated patients. Therefore, the value of these studies has to be considered as very limited. However, most of the included studies reporting on isolated cartilage repair at the patella stated sufficient information regarding inclusion and exclusion criteria. In most of these studies, patients with significant patellofemoral malalignment were excluded based on clinical evaluation or radiological measurements. On the one hand, these strict selection criteria enable a homogeneous study collective and subsequently a good evaluation of the efficacy of the cartilage repair technique itself. On the other hand, however, the study collectives do not represent the majority of patients affected by patellar cartilage defects, considering the high association with anatomic risk factors of up to 88% [44]. The exclusion of patients with patellofemoral malalignment may lead to a distortion of the results because more complex cases were not investigated. This statement can be supported by the fact that the preoperative scores demonstrated higher values among the studies with cartilage repair alone in comparison with the studies including patients with the need for additional procedures (Lysholm score 61.6 vs. 51.9).

Several studies consistently concluded that the avoidance of correcting underlying co-pathologies of retropatellar chondral lesions leads to poorer outcomes [10, 53, 54]. Anatomic abnormalities which have been proven to correlate with cartilage lesions in the patellofemoral joint are trochlea dysplasia, increased TTTG distance, genu valgum and increased femoral antetorsion, while in most cases a combination of these factors is present [3, 21, 43, 44].

Among the 20 studies, including patients who underwent concomitant surgeries, the most frequently performed additional soft-tissue procedures were lateral retinaculum release and MPFL reconstruction, while the most frequently performed bony procedures were osteotomies of the tibial tubercle. All of these techniques have been demonstrated to be successful options to improve patellofemoral alignment[36, 56, 59, 61]. Although trochlea dysplasia has been shown to be one of the most frequent co-pathologies in patients with patellar cartilage defects, trochleoplasty has been performed only in very few cases. A recent study investigated the influence of trochlea dysplasia on the outcome after patellofemoral ACI by means of a comparative matched-pair analysis between 23 patients with high-grade trochlea dysplasia (Déjour types B-D) and 23 patients without trochlea dysplasia [7]. There were no significant group differences regarding clinical outcomes and failure rates after a mean follow-up of 3.7 years. Considering these findings and the rather high invasiveness of the procedure, it can be concluded that the indication for trochleoplasty in combination with cartilage repair at the patella should be set carefully and only in cases with severe patellofemoral instability.

Further identified risk factors for patellofemoral cartilage defects are valgus malalignment and increased femoral antetorsion [21]. However, among all 35 included studies there were no reports on varization or torsional osteotomies in combination with cartilage repair at the patella. Previous studies have demonstrated the efficiency of varization and torsional osteotomies to improve patellofemoral alignment in the field of patellofemoral instability and patellofemoral pain [34, 46, 66]. The clinical evidence of these procedures in combination with cartilage repair is yet to be investigated.

Studies which investigated representative study cohorts also including complex cases with the need for additional procedures showed good results after cartilage therapy at the patella. In comparison with the studies investigating isolated cartilage repair alone, results were similar at the final follow-up. Considering the fact that the mean preoperative scores were lower in the studies including combined procedures, the postoperative benefit may be even larger in this group.

A previous systematic review by Trinh et al. investigated the postoperative outcomes after ACI with or without additional patellofemoral osteotomy [70]. Based on 11 included studies, the authors found greater improvements in clinical scores after combined procedures, which supports the findings of the present systematic review.

This study, as all systematic reviews, has several limitations. First, there was a large heterogeneity of study designs, study qualities, patient population, outcome measurement instruments and data reporting across the included studies. Accordingly, a significant comparison of the individual results of studies with or without respecting co-pathologies such as patellofemoral and femoro-tibial malalignment is limited. Furthermore, due to a probably existing selection bias of included studies of patients treated with patellar chondral repair only, a careful interpretation of the results is required, not allowing for a deductive conclusion. Second, although the included studies reported an adequate overall mean follow-up of at least 50.2 months, the wide range of 24–153-month follow-up of the individual surveys may additionally limit the interpretation of the PROMs. Finally, as a cause of inconsistent documentation, long-term complications reported in some of the studies of this review could be considered.

Despite these limitations, the findings of this systematic review provide clinically relevant information. The results of the included studies demonstrate that the need for simultaneous correction of patellofemoral risk factors leads to similar clinical outcomes in comparison with isolated cartilage repair at the patella. An even larger benefit may be expected for patients with the need for additional procedures.

## Conclusion

This study demonstrated good clinical outcomes after patellar cartilage repair with no evidence of worse results in complex cases with the need for additional patellofemoral realignment procedures. However, a meaningful statistical comparison between isolated patellar cartilage repair and combined co-procedures was not possible due to heterogeneous patient cohorts and a lack of analysis of specific subgroups in recent literature.
